# Potential of recombinant *Mycobacterium paragordonae* expressing HIV-1 Gag as a prime vaccine for HIV-1 infection

**DOI:** 10.1038/s41598-019-51875-6

**Published:** 2019-10-29

**Authors:** Byoung-Jun Kim, Bo-Ram Kim, Yoon-Hoh Kook, Bum-Joon Kim

**Affiliations:** Department of Microbiology and Immunology, Biomedical Sciences, Liver Research Institute and Cancer Research Institute, College of Medicine, Seoul National University, Seoul, Korea

**Keywords:** Live attenuated vaccines, Clinical microbiology

## Abstract

Recombinant *Mycobacterium* strains such as recombinant BCG (rBCG) have received considerable attention for the HIV-1 vaccine development. Recently, we described a temperature-sensitive *Mycobacterium paragordonae* (Mpg) strain as a novel live tuberculosis vaccine that is safer and showed an enhanced protective effect against mycobacterial infection compared to BCG. We studied the possibility of developing a vaccine against HIV-1 infection using rMpg strain expressing the p24 antigen (rMpg-p24). We observed that rMpg-p24 can induce an increased p24 expression in infected antigen presenting cells (APCs) compared to rBCG-p24. We also observed that rMpg-p24 can induce enhanced p24 specific immune responses in vaccinated mice as evidenced by increased p24-specific T lymphocyte proliferation, gamma interferon induction, antibody production and cytotoxic T lymphocyte (CTL) responses. Furthermore, an rMpg-p24 prime and plasmid DNA boost showed an increased CTL response and antibody production compared to rBCG or rMpg alone. In summary, our study indicates that a live rMpg-p24 strain induced enhanced immune responses against HIV-1 Gag in vaccinated mice. Thus, rMpg-p24 may have potential as a preventive prime vaccine in a heterologous prime-boost regimen for HIV-1 infection.

## Introduction

The effective control of HIV replication in infected patients is currently achieved via highly active antiretroviral therapy (HAART). However, the development of effective vaccine is still urgent to prevent the viral infection due to several problems, including the drug resistant variants and the high cost by long term treatment^1^.

Recently, due to its proven safety and excellent adjuvant properties, *Mycobacterium bovis* bacille Calmette-Guérin (BCG), the only live attenuated vaccine used to protect against tuberculosis (TB), is being studied as a live vaccine vector to confer protection against infectious pathogens and cancer. Recombinant BCG (rBCG) has been constructed to express antigens of TB^2,3^ or other pathogens, including those from *Borrelia burgdorferi*^4^, *Bordetella pertussis*^5^, *Streptococcus pneumoniae*^6^, human immunodeficiency virus type 1 (HIV-1)^7^, measles virus^8^, simian immunodeficiency virus (SIV)^9^, leishmania^10^, rodent malaria^11^. However, vaccine protocols using rBCG has not generated considerable protection for the target infection, as observed in the failure of a Lyme disease vaccine in human clinical trials^12^. The disappointing protection generated using the rBCG vaccine may be due to the unstable expression or inefficient antigen processing and presentation of foreign genes. Furthermore, the immune system modulating nature of BCG itself, including the promotion of lower levels of IL-12 secretion or enhanced ERK dependent IL-10 secretion, could result in a failure to elicit sufficient protective immunity in vaccine applications^13^. Thus, to overcome the problems of rBCG for vaccine development, several approaches using recombinant *M. smemgatis* (rSmeg) instead of BCG have been introduced^14,15^. However, the results of our previous study comparing rBCG and rSmeg expressing HIV-1 Gag p24 proteins to induce protective immune responses in vaccinated mice indicated the superiority of rBCG over rSmeg, particularly in the induction of p24-specific humoral immune responses^16^. Thus, the selection of an alternative mycobacterial strain to guarantee increased protective immune responses compared to rBCG is needed for vaccine development of infectious agents, inducing HIV-1.

Recently, we introduced a temperature sensitive mycobacterial species, *Mycobacterium paragordonae* (Mpg), which cannot grow at 37 °C, indicating its potential in the development of a safe, temperature sensitive vaccine for tuberculosis infection instead of BCG^17^. Indeed, our further studies described that compared to BCG, Mpg was safer in infected cells and mice and exerted an enhanced protective effect against *M. tuberculosis* or even *M. abscessus* infection^18^ demonstrating the potential of rMpg for vaccine development as an alternative to rBCG. To explore this possibility, in the present study, we first developed an rMpg-p24 strain expressing HIV-1 Gag p24 proteins and compared its capacity to induce p24-specific immune responses with rBCG-p24 in mice vaccinated with mycobacteria alone. In addition, we explored whether the p24-specific immune response elicited by rMpg-P24 could be augmented by boosting with a p24- encoding plasmid DNA vaccine.

## Results

### rMpg-p24 exhibits increased p24 expression in infected bone marrow derived dendritic cells (BMDCs) compared to rBCG-p24

**Figure 1:**
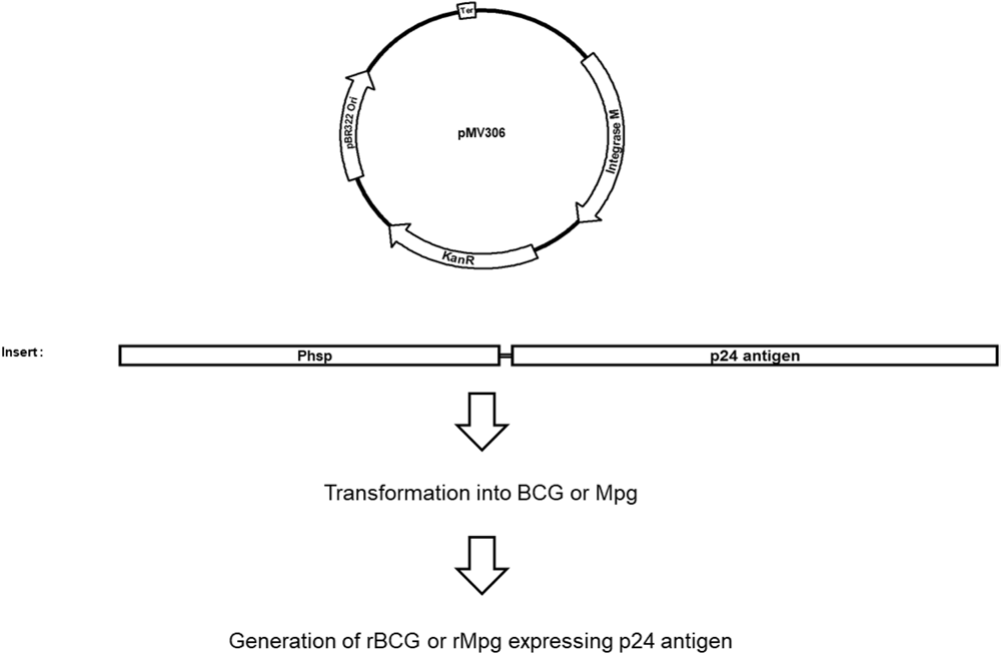
Maps of p24 expression vectors used in  this study. Maps of the constructed *Mycobacterium-E. coli* shuttle vectors for p24 expression. pMV306-p24 vector can express p24 under control of the *hsp65* promoter from *M. bovis* BCG.

**Figure 2:**
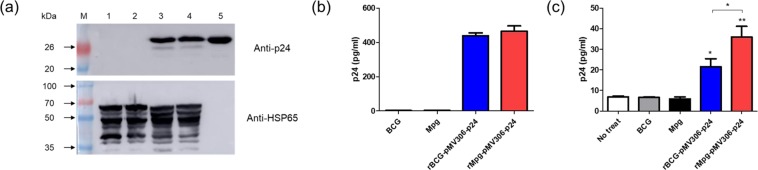
Expression levels of p24 in rBCG or rMpg strain and in cell lines infected with rMpg or rBCG strain. (**a**) Confirmation of p24 expression in rMpg or rBCG strain using a western blot analysis. Proteins were extracted from wild type BCG (lane 1), Mpg (lane 2), rBCG-p24 (lane 3), or rMpg-p24 (lane 4) strains. Purified p24 protein was used as a positive control (lane 5). M, molecular weight standard (Elpis Bio, Taejeon, Korea; DokDo-MARK^TM^). Mycobacterial HSP65 was also confirmed as an internal control. Distinct membranes were separated by white space. The western blot image was cropped from a full-length blot for improving the clarity and conciseness. The full-length blot image is presented in Supplementary Fig. S3. (**b**) Confirmation of p24 expression in rBCG-p24 or rMpg-p24 strain using ELISA. (**c**) The expression levels of p24 after the infection of the BMDCs with wild-type BCG, Mpg, rBCG-p24 or rMpg-p24 strains. Data are representative of two independent experiments. Means ± SD are shown. *P < 0.05; **P < 0.01 (Student’s *t*-test).

To examine the usefulness of rMpg for HIV-1 p24 Gag vaccination, we generated an rMpg strain expressing p24, rMpg-p24, using the integrative *Mycobacterium-E. coli* shuttle vector pMV306^19^ (Fig. 1). The growth rates of the wild-type Mpg and rMpg-p24 strains in 7H9 broth (with 100 μg/ml of kanamycin) cultured for 14 days were compared and showed almost identical growth rates (Supplementary Fig. S1). To compare the p24 expression in the rBCG- and rMpg-p24 strains, we conducted western blot (Fig. 2a) and ELISA (Fig. 2b) analyses against p24 using bacterial lysates. Both recombinant strains expressed almost the same level of p24 protein. To test the p24 stability in rMpg-p24 strain, the p24 levels in rMpg-p24 after various numbers of passages on 7H10 agar plates with or without kanamycin were also determined by ELISA. The rMpg-p24 strain stably expressed p24, even after 12 passages on the 7H10 agar plates with or without kanamycin (Supplementary Fig. S2). Additionally, we examined the p24 levels by ELISA in bone-marrow derived dendritic cells (BMDCs) infected with the rBCG-p24 and rMpg-p24 strains. The result showed that the p24 level in the rMpg-p24-infected BMDCs was higher than that observed in cells infected with rBCG-p24 (Fig. 2c). Taken together, our results indicate that the developed rMpg-p24 strain showed stable p24 expression within the recombinant bacteria and led to an enhanced production of p24 protein in infected BMDCs compared to that observed in cells infected with rBCG-p24.

### BMDCs infected with rMpg-p24 exhibit increased T cell proliferation in mice immunized with p24 antigen

**Figure 3:**
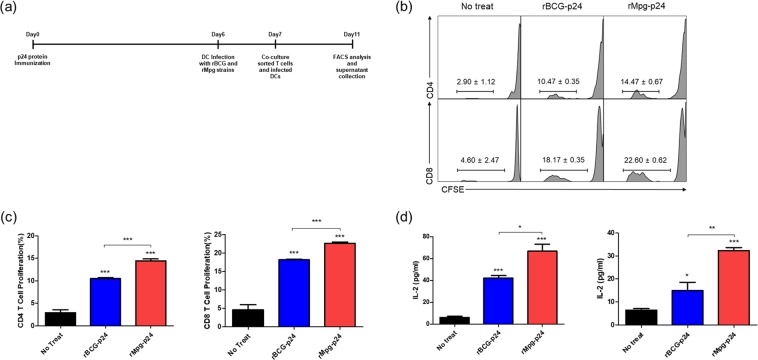
T cell proliferation levels induced by BMDCs infected with the rBCG-p24 or rMpg-p24 strain. (**a**) Schematic schedule of the T cell proliferation assay. Two mice were injected with p24 protein (30 μg/mouse) and after 7 days, their splenocytes were sorted into CD4 and CD8 T cells and labeled with CFSE. One day before co-cultivation, DCs were infected with each strain (10 M.O.I.). Four days after co-cultivation of CFSE-labeled CD4/CD8 T cells and infected DCs, cells were analyzed for T cell proliferation (**b**,**c**). Flow cytometric analysis of the proliferation of CFSE-labeled CD4 and CD8 T cells following the infection of BMDCs with the rBCG-p24 or rMpg-p24 strains. (**d**) ELISA determination of IL-2 in the supernatants of CD4 (left panel) or CD8 (right panel) cells co-cultured with infected BMDCs. Data are representative of three independent experiments. Means ± SD are shown. *P < 0.05; **P < 0.01; *** *P* < 0.001 (Student’s *t*-test).

To determine whether rMpg-p24 induces T cell proliferation against the p24 antigen, we carried out a T cell proliferation assay by co-culturing BMDCs infected with two recombinant strains, either rBCG-p24 or rMpg-p24, and sorted T cells exposed to p24 antigen using CFSE dilution methods^20^. Figure 3a showed the schedule for the T cell proliferation assay. rBCG-p24 or rMpg-p24 strain infected BMDCs elicited significantly higher levels of T cell proliferation than the un-infected BMDCs. Notably, the BMDCs infected with rMpg-p24 induced significantly higher levels of T cell proliferation than those infected with the rBCG-p24 strain (Fig. 3b,c). IL-2 cytokine levels from co-cultured supernatant also supported the results of T cell proliferation assay (Fig. 3d). Taken together, these results suggest that the use of the rMpg-p24 strain led to enhanced p24-specific T cell proliferation activity in vaccinated mice compared to that observed with the rBCG-p24 strain.

### rMpg-p24 elicits an enhanced generation of p24-specific IFN-γ spot forming cells (SFC) in splenocytes from immunized mice

**Figure 4:**
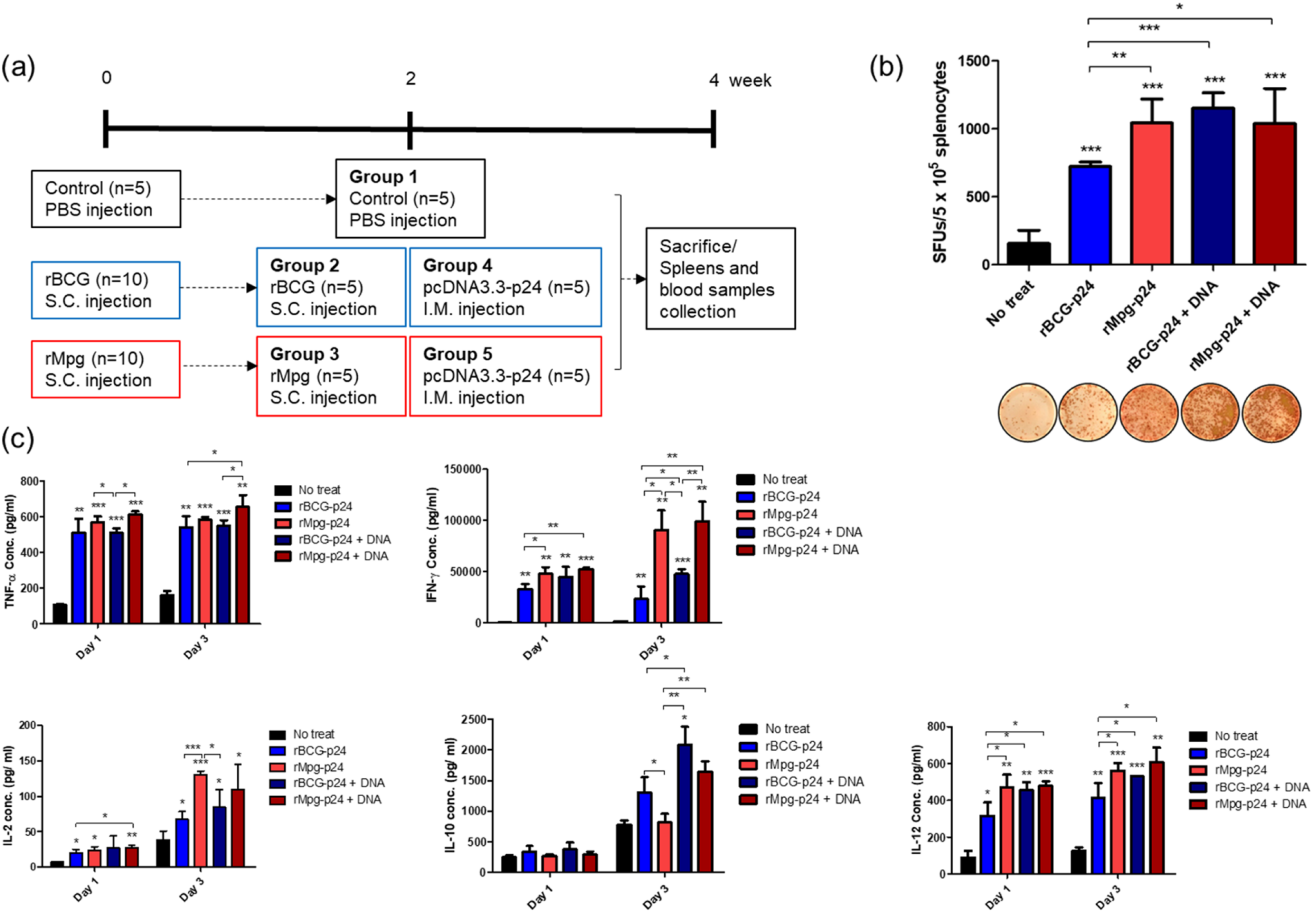
The five different vaccination groups used in this study and comparison of immune responses between different vaccination groups. (**a**) Schematic representation of five different vaccination groups used in this study.  With two weeks interval, mice of groups 2 and 3 (five mice/group) were immunized twice with rBCG-p24 or rMpg-p24 strains, alone. Mice of groups 4 and 5 were immunized first with rBCG-p24 or rMpg-p24 strain and then immunized with pcDNA3.3-p24 DNA as boost vaccine. Two weeks after final immunization, mice were sacrificed and their spleens and blood samples were collected for immunological analyses. (**b**) IFN-γ secretion levels following *in vitro* stimulation of splenocytes from mice vaccinated with the rBCG-p24 or rMpg-p24 strain, alone or prime vaccination with recombinant strains and boosting with pcDNA3.3-p24 DNA were detected using an ELISPOT analysis. Representative images of ELISPOT membrane in each group are shown below the graph. (**c**) Levels of the IL-2, IFN-γ, TNF-α, IL-6 and IL-10 cytokines following *in vitro* stimulation with p24 of splenocytes from mice vaccinated with the rMpg-p24, rBCG-p24 strains and/or with pcDNA3.3-p24 DNA were detected using ELISA analyses. A total of 5 mice per group was analyzed. Data are representative of two independent experiments. Means ± SD are shown. *P < 0.05; **P < 0.01; ***P < 0.001 (Student’s *t*-test).

To determine whether heterologous prime-boost vaccination regimens using rMpg-p24 as prime vaccination and plasmid DNA as boosting immunogen improved the p24-specific immune response, splenocytes were isolated from the spleens of BALB/c mice (five mice/group) subcutaneously (s.c.) immunized twice with rBCG-p24 (Group 2) or rMpg-p24 (Group 3) or mice immunized with prime-boosting protocol of an i.m. injection of heterologous pcDNA3.3-p24 plasmid (Group 4, rBCG-p24+ pcDNA3.3-p24; and Group 5, rMpg-p24+ pcDNA3.3-p24) (Fig. 4a) and assayed for detecting the p24-specific IFN-γ producing cells using IFN-γ ELISPOT assays. The splenocytes from the mice immunized twice with rMpg-p24 strain (Group 3) produced significantly higher IFN-γ SFUs than those from the mice immunized twice with the rBCG-p24 strain (Group 2), suggesting the superiority of the rMpg-p24 vaccination over rBCG-p24 in inducing p24-specific cell-mediated immunity (CMI). The splenocytes from mice immunized with rBCG-p24 as a prime vaccination and boosted with pcDNA3.3-p24 DNA (Group 4) showed higher SFUs than those from the mice immunized only with the rBCG strain, suggesting that the use of a DNA boosting strategy with the rBCG-p24 strain could lead to additive effect for inducing a p24-specific CMI response. However, no significant difference was observed between Groups 3 and 5 in p24-specific IFN-γ SFUs from vaccinated mice, suggesting that there may be no additive effect of the heterologous prime-boost strategy when using rMpg-p24 as a prime vaccination with respect to inducing a p24-specific CMI response (Fig. 4b). This result may be because rMpg could lead to a strong and saturating CMI response without the DNA boosting vaccination. Taken together, our results indicate that the rMpg-p24 strain induced an increased p24-specific production of IFN-γ, which represents a Th-1 signature cytokine, compared to that observed with the rBCG-p24 strain, suggesting its possibility for improving the efficacy of the vaccine by skewing Th-1 type immune responses.

### rMpg-p24 induces an increased production of Th-1 or pro-inflammatory cytokines in splenocytes from immunized mice

Cytokine levels of IL-2, IFN-γ, TNF-α, IL-10, and IL-12 were detected from the supernatants of *in vitro* stimulated splenocytes with p24 protein (5 μg/ml).

For IL-2 and IFN-γ, Groups 3 and 5 produced higher levels of cytokines in splenocytes at day 3 than the Groups 2 and 4 (Fig. 4c and Supplementary Table S1). In the case of TNF-α, Groups 3 and 5 produced slightly higher cytokine levels than Groups 2 and 4. Regarding the IL-12 cytokine levels, Groups 3 and 5 also showed slightly higher expression levels than Groups 2 and 4, suggesting that rMpg can lead to an enhanced production of inflammatory and Th1-type cytokines in splenocytes from vaccinated mice. However, Group 2 produced higher levels of IL-10 than Group 3, while Group 4 produced higher levels than Group 5, suggesting that rMpg can lead to decreased levels of Th2-type cytokines in splenocytes from vaccinated mice (Fig. 4c and Supplementary Table S1). For IL-10 production at day 3, a significant difference between Groups 3 and 5 was observed, suggesting an additive effect of the DNA boosting strategy on IL-10 production in the rMpg-p24 vaccine protocol. However, for other cytokines, significant differences between the two groups were not observed.

### rMpg-p24 vaccination and DNA boosting induces an p24-specific Th1-biased humoral immune response in immunized mice

**Figure 5:**
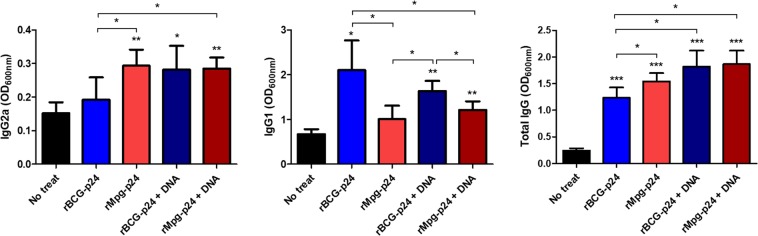
Comparison of  humoral immune responses induced between different vaccination groups. p24 specific immunoglobulin subtypes (IgG2a, IgG1 and total IgG) were detected by ELISA at 450 nm. OD values for the IgG2a and IgG1 subtypes and the total IgG levels were compared. Serum samples from five mice per group were analyzed. Data are representative of two independent experiments. Means ± SD are shown. *P < 0.05; **P < 0.01; ***P < 0.001 (Student’s *t*-test).

We analyzed the p24-specific IgG2a and IgG1 isotypes, which are known markers of Th1 and Th2 responses^21–23^, to determine whether the rMpg-p24 vaccination and/or DNA boosting induced a Th1-biased humoral immune response in immunized mice. As shown in Fig. 5, significantly higher levels of p24-specific IgG2a isotype were observed in Group 3 than in Group 2, suggesting that rMpg-p24 induced an increased Th1-biased humoral response compared to rBCG-p24. However, no significant difference was observed between Groups 3 and 5, suggesting that in the rMpg-p24-based vaccine protocol, the DNA boosting strategy did not lead to additive effect for inducing p24-specific IgG2a production. Regarding the IgG1 isotype, Groups 3 and 5 showed significantly lower levels of the IgG1 isotype than Groups 2 and 4. For total IgG, Group 3 showed significantly higher IgG levels than Group 2, and Groups 4 and 5 showed the highest IgG expression levels (Fig. 5). Consistently, the IgG2a/IgG1 ratio, where a higher ratio means a greater Th1-biased immune response^22^, was significantly higher in Groups 3 and 5 (1.38 ± 0.52 and 1.05 ± 0.16, respectively) than that observed in Groups 2 and 4 (0.48 ± 0.34 and 0.76 ± 0.11, respectively), suggesting that rMpg-p24 induced an increased p24-specific Th1-biased humoral immune response in immunized mice.

### rMpg-p24 vaccination and DNA boosting induced an increased p24-specific cytotoxic T lymphocyte response in immunized mice

**Figure 6:**
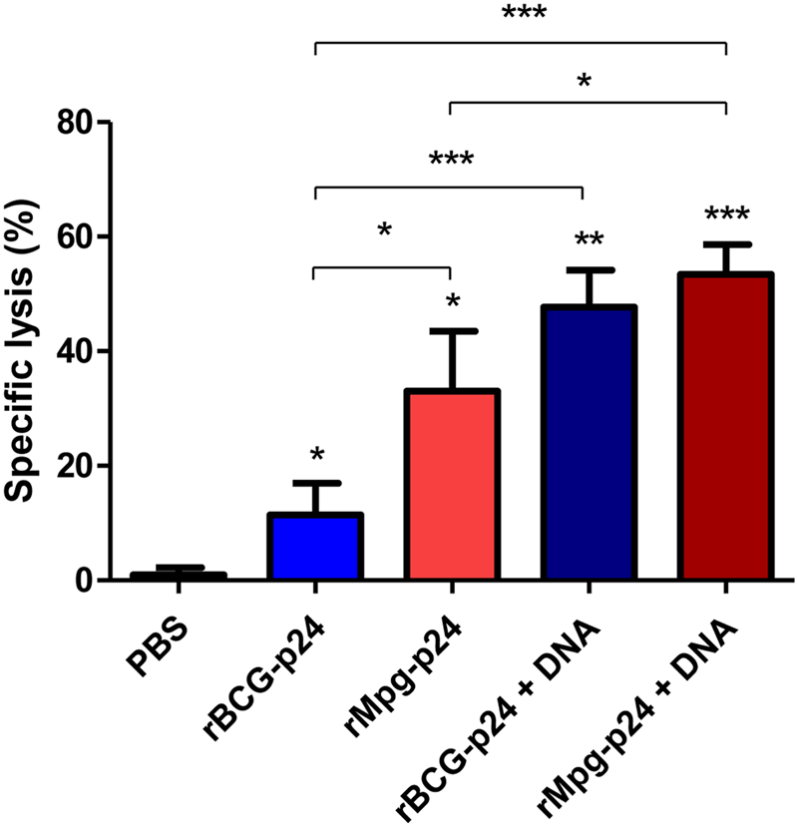
Comparison of  cytotoxic T lymphocyte responses induced between different vaccination groups.  CTL responses from mice subject into different vaccine protocols were analyzed from stimulated splenocytes with p24 (effector cells) and p24 epitope peptide (A9I) pulsed P815 cells (target cells). Three mice per group were analyzed. Data are representative of two independent experiments. Means ± SD are shown. *P < 0.05; **P < 0.01; ***P < 0.001 (Student’s *t*-test).

To determine whether the rMpg-p24 vaccination and DNA boosting induced an increased p24-specific cytotoxic T lymphocyte (CTL) response in immunized mice, we analyzed the CTL activity in co-cultured P815 cells and splenocytes from Groups 1 to 5 via an LDH cytotoxicity assay. The immunization procedure is described in Fig. 4a. A9I peptide pulsed P815 cells (H-2^d^) were used as the target cells, and the effector/target ratio was 50:1, as previously described^24^. As shown in Fig. 6, the CTLs in the Group 3 mice exhibited a significantly higher level of p24-specific target cell lysis than those from Group 2, suggesting the superiority of rMpg-p24 over rBCG-p24 in eliciting a p24-specific CTL response (Fig. 6). The DNA boosting groups (Groups 4 and 5) showed significantly enhanced CTL responses compared to the groups injected with the recombinant mycobacterial strains alone (Groups 2 and 3), suggesting an additive effect of DNA boosting for eliciting CTL response, as was previously reported^25^ (Fig. 6).

### Multifunctional T cell responses induced by rMpg-p24 vaccination and DNA  boosting groups

**Figure 7:**
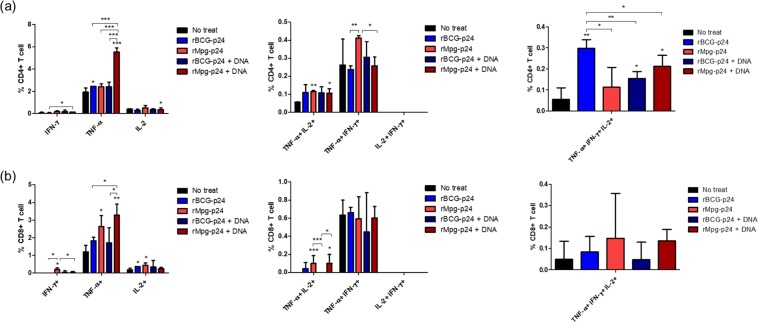
Comparison of  multifunctional T cell responses induced between different vaccination groups.  Cell population of (**a**) CD4 T cells and (**b**) CD8 T cells showing distinct  patterns of cytokine production (TNF-α+, IFN-γ+ and IL-2+) was compared between different vaccine groups. Three mice per group were analyzed. Data are shown with Means ± SD in triplicate. *P < 0.05; **P < 0.01; ***P < 0.001 (Student’s *t*-test).

A multifunctional T cell response has been reported to play a pivotal role in the protection against infectious agents, including *M. tuberculosis*^26,27^. Therefore, we compared expression of IFN-γ, TNF-α, and IL-2 in CD4 and CD8 T cell populations from mouse splenocytes in Groups 1 to 5 after stimulation with p24 antigen (10 μg/ml). Responding CD4 and CD8 T cells were classified as triple (3+; IFN-γ+ TNF-α+ IL-2+), double (2+; TNF-α+ IL-2+, TNF-α+ IFN-γ+ and IL-2+ IFN-γ+) or single (1+; TNF-α+, IL-2+ and IFN-γ+) cytokine-producing populations according to the IFN-γ, TNF-α, and IL-2 cytokine expression profiles. No significant differences in the single positives of IFN-γ+ and IL-2+ CD4 T cell populations were observed among the immunized groups. However, for the TNF-α+ CD4 T cells, Group 5 exhibited the greatest number of TNF-α positive cells compared to the other groups. For the TNF-α+ IFN-γ+ double positive CD4 T cells, Group 3 had the greatest number of cells among the assayed groups. In the p24-specific triple positive CD4 T cells, significantly more cells were observed in Group 2 than in Groups 3 to 5. A greater number of triple positive CD4 T cells from Group 5 were observed compared to Group 3, despite it did not reach significant level (Fig. 7a).

In the single positive CD8 T cells, the greatest number of IFN-γ+ single CD8 T cells were observed in Group 3 compared to the other groups. Greater numbers of TNF-α+ single positive CD8 T cells were observed in Groups 3 and 5 than in Groups 2 and 4. Interestingly, greater numbers of TNF-α+ IL-2+ double positive CD8 T cells were observed Groups 3 and 5 than in Groups 2 and 4. However, no significant differences in the populations of TNF-α+ IFN-γ+ double positive and IFN-γ+ TNF-α+ IL-2+ triple positive CD8 T cells were observed among the immunized groups (Fig. 7b). Collectively, the results showed that the rMpg-p24 vaccine protocol led to enhanced p24-specific TNF-α-producing single or multifunctional CD4 and CD8 T cell populations, which could contribute to an enhanced p24-specific immune response.

## Discussion

In the present study, we demonstrated that rMpg-p24 expressing HIV-1 Gag p24 proteins could lead to enhanced HIV-1 p24-specific immune responses in vaccinated mice via two protocols, one involving twice s.c. injections of rMpg-p24, alone and the other involving a heterologous prime-boost vaccination regimen using rMpg-p24 as the prime and plasmid DNA as the boost (Fig. 4a). The DC loading of p24 antigen (Ag) (Figs 2c and 3), CMI responses, such as the levels of IFN-γ and Th1-type cytokine production (Fig. 4) and Th1-biased humoral immune responses (Fig. 5) and the p24-specific CTL (Fig. 6) were robustly enhanced in mice vaccinated using the rMpg-p24 protocol versus rBCG-p24. In addition, significantly higher levels of p24-specific TNF-α-producing single or multifunctional CD4 and CD8 T cell populations were also observed (Fig. 7) in mice vaccinated with rMpg-p24.

There are some noteworthy advantages of rMpg-p24 over rBCG-p24 as a vaccine for protection against HIV-1 infection. First, rMpg-p24 is a safer *in vivo* vaccine challenge as a live vaccine compared to rBCG-p24. The results of our previous study on the potential of Mpg as a tuberculosis vaccine^18^ indicated that a temperature-sensitive Mpg, which cannot grow at the core body temperature, was safer in an *in vivo* challenge as a live vaccine than rBCG, providing a rationale for the development of safe live vaccine using rMpg-p24. Indeed, we also observed that rMpg-p24 was also safer in a mice challenge model compared to rBCG-p24 (data not shown). Second, rMpg-p24 led to an enhanced Ag delivery into DCs and enhanced DC maturation. Our data showed that although the amount of p24 expressed by bacteria was almost similar between the two recombinant bacteria, the amount of p24 delivered into BMDCs by rMpg-p24 was higher than that observed for rBCG-p24 (Fig. 2c), which could enhance the levels of p24-derived peptides presented to T cells. Indeed, our T cell proliferation study showed that rMpg-p24 versus rBCG-p24 elicited enhanced CD4 or CD8 T cell proliferation (Fig. 3). Furthermore, the results of our previous study indicated that Mpg also led to significantly enhanced DC maturation compared to BCG^18^. Taken together, the ability of rMpg to deliver a higher level of p24 Ag into DCs and elicit DC maturation could contribute to an enhanced adaptive immune response against HIV-1 p24. Third, compared to rBCG-p24, rMpg-p24 could exert an enhanced ratio of IL-12 to IL-10 in splenocytes from vaccinated mice (Fig. 4c), a signature marker for skewing into the Th1 immune response, which was similar to the findings of our previous study^18^. Fourth, our IFN-γ ELISPOT and cytokine production assay showed that compared to rBCG-p24, the use of rMpg-p24 led to enhanced p24-specific IFN-γ production from splenocytes of vaccinated mice, which is master cytokine for the Th1 adaptive immune response (Fig. 4b,c). The enhanced rMpg-p24-induced IFN-γ production could induce p24-specific adaptive immune response via increased antigen presentation of p24 to T cells by activation of APCs, such as DCs or macrophages. Fifth, our data showed that compared to rBCG-p24, the use of rMpg-p24 led to enhanced vaccine effector functions, a p24-specific Th1-biased humoral response (Fig. 5) and enhanced CTL responses (Fig. 6), contributing to an enhanced protective effect against HIV-1 infection. Finally, our multifunctional T cell analysis showed that the use of rMpg-p24 led to enhanced TNF-α-producing single positive or multifunctional T cell populations, including TNF-α single positive CD4 T cells, TNF-α+ IFN-γ+ double positive CD4 T cells and TNF-α single positive CD8 T cells in the rMpg vaccination or rMpg and DNA boosting vaccination groups (Fig. 7). Given the previous reports that TNF-α-producing T cells can play a pivotal role in vaccine-mediated protection of cancer^28^, HBV infection^29,30^ or protection against *Leishmania major*^31^, it is possible that p24-specific TNF-α-producing T cells induced by rMpg contribute to an enhanced vaccine efficacy.

In addition, we sought to assess the additive effect of a boosting DNA vaccine on p24-specific immune responses in an rMpg prime and DNA boost vaccine protocol. Unlike the rBCG prime and DNA boost vaccine protocol, which showed an additive effect of the DNA boosting vaccine on most of p24-specific immune responses, including IFN-γ production, Th1-type cytokine production (Fig. 4b,c), and Th1-biased humoral immune responses (Fig. 5) and the CTL response (Fig. 6), only the CTL response and total IgG amounts were  induced by the DNA boosting vaccination in the rMpg prime and DNA boost vaccine protocol. The difference between the two protocols with respect to the role of DNA boosting may be attributed to differences between the two recombinant strains used for the prime vaccine, rMpg and rBCG, in their capacity to elicit p24-specific immune responses when first used for vaccination as described above. In further studies, we plan to examine the value of immune response induced by priming DNA first and boosting with rMpg-p24 strain.

The major aim of this study is to prove the usefulness of rMpg for delivery system of HIV vaccine target Ags. So, in the future, we have plans to select proper HIV antigens other than p24 to be delivered by rMpg system via considering the immune correlates of risk as reported previously^32,33^. And then, rMpg expressing HIV antigens finally selected would be assessed via *in vivo* infection system using non-human primates for further vaccine development. In summary, we demonstrated that the rMpg-p24 strain developed in this study delivered more p24 antigen into phagocytes than the rBCG-p24 strain. In addition, we showed that the rMpg-p24 strain could enhance the T cell proliferation capacity of infected BMDCs and elicit improved CTL and Th1-biased humoral immune responses in vaccinated mice, compared to rBCG-p24. In addition, we also observed an additive effect of DNA boosting on the p24-specific CTL response in the rMpg prime and DNA boost vaccine protocol. Our results suggest a great potential of rMpg-p24 for use in the vaccination against HIV-1 infection as a live attenuated vaccine or as prime vaccine for a heterologous prime-boost strategy. In further studies, we plan to evaluate the rMpg as an alternative to rBCG for *in vivo* vaccine delivery of diverse foreign genes, such as HIV antigens, for the development of preventive vaccine for various infectious diseases.

## Materials and Methods

### Mice and immunization procedures

Female BALB/c mice (~25 g, 7 weeks old) were purchased from Orient-Bio (Seoul, Korea) and used in experiments at 8 weeks of age. The mice were randomly divided into four groups of five mice per group.

For T cell proliferation assays, the p24 protein was injected into two mice (BALB/c) through the tail vein (30 μg/mouse), and five mice (BALB/c) were used to prepare bone-marrow derived dendritic cells (BMDCs) in each test.

For vaccination tests, the BALB/c mice were subcutaneously (s.c.) immunized twice with i) the rBCG-p24 (Group 2) or ii) the rMpg-p24 (Group 3) (1 × 10^6^ CFU in 100 μl PBS at a 4-week interval) at the bottom of the tail. The DNA boosting groups were injected with the pcDNA3.3-p24 expression plasmid (30 μg/mouse) via the intramuscular (i.m.) route two weeks after one s.c. injection of iii) the rBCG-p24 (Group 4) or iv) the rMpg-p24 (Group 5). For the negative control group (Group 1), PBS was injected subcutaneously. Two weeks after the final immunization, the mice were euthanized at each time point by CO_2_ inhalation, and their blood and spleens were removed and used in the immunological assays, such as IFN-γ ELISPOT, cytokine determination, serum antibody detection (five mice/group), CTL analysis (three mice/group) and multifunctional T cell analysis.

### Ethics statement

All animal experiments were carried out in accordance with the recommendations of the institutional guidelines and the protocol was approved by the Institutional Animal Care and Use Committee (IACUC; approval No. of SNU-141201-3-1) of the Institute of Laboratory Animal Resources at Seoul National University.

### Generation of an rMpg expressing HIV-1 p24 Gag

To generate the rMpg expressing HIV-1 p24 Gag, the pMV306-p24 plasmid^24^ was electroporated into competent Mpg strain cells (JCM 18565^T^) using a Gene Pulser II electroporation apparatus (Bio-Rad, Hercules, CA, USA)^34^. Typically, colonies of transformants were selected from the plates, transferred into 7H9 broth medium (Difco Laboratories, Detroit, MI, USA) supplemented with 0.5% glycerol, 0.05% Tween-80, 10% ADC and 100 μg/ml of kanamycin and were cultured for 2 weeks at 30 °C. The growth rate of the rMpg strain was determined by measuring the optical density (OD) at 600 nm. The rBCG-p24 strain, which was generated as previously described^16^, was used and compared to the rMpg-p24 strain generated in this study.

### Generation of bone marrow-derived dendritic cells from mice

Bone marrow derived dendritic cells (BMDCs) were generated from the bone marrow (BM) of 8- to 12-week-old BALB/c mice as previously described^35^. Briefly, the BM cells were differentiated with complete IMDM supplemented with 10% FBS (Gibco Invitrogen), recombinant mouse GM-CSF (1.5 ng/ml; PeproTech, Rocky Hill, NJ, USA) and mouse IL-4 (1.5 ng/ml; PeproTech, USA), penicillin (100 units/ml; Gibco Invitrogen), streptomycin (100 μg/ml; Gibco Invitrogen), gentamicin (50 μg/ml; Gibco Invitrogen), L-glutamine (2 mM; Gibco Invitrogen), and β-mercaptoethanol (50 nM; Gibco Invitrogen). Five mice were used to prepare BMDCs for each experiment, and five 24 well plates were used for differentiating the BMDCs.

### Determination of the p24 Gag expression levels in the rMpg-p24 strain

To determine the p24 Gag expression levels in the rMpg or rBCG strain, we conducted western blot and enzyme-linked immunosorbent assay (ELISA) analyses as previously described^24^. The expression of p24 in the rMpg-p24 or rBCG-p24 strain was determined using a mouse anti-p24 monoclonal antibody (Abcam, Cambridge, USA; 1:1,000 dilution). As an internal control, mycobacterial HSP65 (Abcam, 1:1,000 dilution) was used. To assess the stable expression of p24, the p24 expression level in the rMpg-p24 strain after various passages (after 1, 4, 6, 8, 10 and 12 passages) was also determined. The passage process was conducted from plate to plate (7H10 agar plate with or without kanamycin), and the colonies from each passage were cultured in 7H9 broth medium for 2 weeks prior to performing each experiment. Additionally, the same amount of protein was used to detect the p24 levels using a p24 ELISA kit (in triplicate wells) (ABL, Rockville, USA) as suggested by the manufacturer^36^. All the groups were analyzed in two independent experiments.

### Determination of the p24 Gag expression levels in BMDCs infected with the rMpg-p24 strain

For the rMpg-p24 strain infection assay, 5~10 × 10^5^ BMDCs were seeded per well (24-well plate, in triplicate) and cultured for 18 hr. The BMDCs were infected with the rBCG- or rMpg-p24 strain at a multiplicity of infection (M.O.I) of 10 for 24 hours. To assess the p24 expression in the cells, the total proteins in the cell pellets were prepared by suspension in RIPA lysis buffer and used to assess the p24 levels using a p24 ELISA kit (ABL) (in duplicate wells) according to the manufacturer’s instructions. All of the infection groups were analyzed in duplicate in each experiment, and two independent experiments were conducted.

### T cell proliferation assay

CD4− and CD8-positive T cells from mice immunized with p24 protein (intravenously injection, 30 μg/mouse)^25^ and BMDCs infected with 10 M.O.I. of rBCG or rMpg strain were used. The schematic schedule for the T cell proliferation assay is described in Fig. 3a. Proliferation assays were conducted with carboxyfluorescein diacetate succinimidyl ester (CFSE), CD4 BV421-conjugated anti-CD4 (Clone GK1.5, BD Horizon) and PE-conjugated anti-CD8a (Clone 53-6.7, eBioscience) as described previously. The cell proliferation was determined using a FACS LSRFortessa instrument (BD Biosciences) and were analyzed using Flowjo (Fig. 3a). All of the experiments were conducted in triplicate.

### IL-2 ELISA

The amounts of murine IL-2 in the cocultured supernatants (in triplicate wells) from the above T cell proliferation assay were also determined using ELISA according to the manufacturer’s instructions (BioLegend, USA). All of the experiments were conducted in duplicate.

### IFN-γ Enzyme-Linked Immuno-Spot (ELISPOT) assay

Splenocytes from immunized mice (five mice/group) were used to conduct an ELISPOT assay as previously described^37^. The IFN-γ ELISPOT assay was performed using the mouse IFN-γ capture antibody (3 μg/ml, clone: AN-18) (BD-Biosciences, San Diego, CA, USA) and biotin-labeled mouse IFN-γ detection antibody (3 μg/ml, clone: XMG1.2) (BD-Biosciences). Splenocytes (5 × 10^5^ cells/well) were analyzed 24 hour after re-stimulation with p24 protein at a final concentration of 5 μg/ml. The number of spot forming units (SFUs) per well was automatically counted using an ELISPOT reader (AID ELISPOT reader, Strasburg, Germany). All the groups were analyzed in triplicate, and two independent experiments were conducted.

### Determination of cytokine production in immunized mice

The splenocytes from the immunized mice (five mice/group) were adjusted to a concentration of 1 × 10^6^ cells/well (96-well microplate, 200 μl volume, in triplicate) in RPMI 1640 medium supplemented with 10% FBS, and purified p24 protein was added at a concentration of 5 μg/ml for the *in vitro* stimulation. The cells were cultured, and the supernatants were harvested to determine the levels of the cytokines IL-2 (BioLegend, San Diego, CA, USA), TNF-α (eBioscience, San Diego, CA, USA), IL-10 (eBioscience), IL-12 (eBioscience) and IFN-γ (BioLegend) using ELISA kits. All of the groups were analyzed in triplicate, and two independent experiments were conducted.

### Serum antibody detection

To detect the serum antibody ratio, serum samples were collected by cardiac puncture method from the immunized mice (five mice/group) after euthanasia via CO_2_. The 96-well plate was coated overnight at 4 °C with purified p24 protein (5 μg/ml) in 0.05 M carbonate-bicarbonate buffer (pH 9.6). Serum antibodies were detected using mouse IgG2a, IgG1 (BD Biosciences, 1:1,000 dilution) and total IgG (eBioscience, 1:1,000 dilution) antibodies as described previously^25^. The optical density (OD) was determined using a spectrometer at a wavelength of 450 nm^38^.

### Cytotoxic T lymphocyte (CTL) assay

The induced CTL responses were determined as previously described^39^ with slight modifications. In brief, for the effector cells, the splenocytes from the mice in each immunized group were pulsed using the major histocompatibility complex (MHC) class I-restricted p24 peptide A9I (AMQMLKETI) (10 μg/ml; Peptron, Daejeon, South Korea)^40^ and incubated for six days with IL-2 (30 U/ml; PeproTech, Rocky Hill, USA) at 37 °C in a 5% CO_2_ incubator. The target cells, i.e., P815 cells (H-2^d^), were prepared by an incubation with the A9I peptide (10 μg/ml) for 2 hrs before being cocultured with the effector. Cell cytotoxicity was evaluated using a lactate dehydrogenase (LDH) assay in U bottom 96-well plates according to the manufacturer’s protocol (CytoTox 96 Non-Radioactive Cytotoxicity Assay; Promega, Madison, USA) as described previously.

### Multifunctional T cell responses

Approximately 2 × 10^6^ splenocytes from immunized mice were seeded into 96-well plates in complete RPMI-1640 medium and stimulated with p24 antigen (10 μg/ml) in a total volume of 200 μl for 8 hrs. Stimulation with PMA (50 ng/ml) and ionomycin (1 μg/ml) was used as a positive control. Brefeldin A (10 μg/ml) was added, and the cells were cultured for an additional 4 hrs. Cell surface markers were stained with BV421-conjugated anti-CD3 (Clone: 17A2, BD Horizon^TM^), V500-conjugated anti-CD4 (Clone: RM4-5, BD Horizon^TM^), and PE-Cy5-conjugated anti-CD8a (Clone: 53-6.7, BD Pharmingen^TM^). After permeabilization (Cytofix/Cytoperm kit; BD Pharmingen), intracellular staining was performed using APC-conjugated IFN-γ (Clone: XMG1.2, BD Pharmingen^TM^), FITC-conjugated TNF-α (Clone: MP6-XT22, BD Pharmingen^TM^), and PE-conjugated IL-2 (Clone: JES6-5H4, BD Pharmingen^TM^). Stained cells were analyzed using a FACS LSRFortessa instrument (BD Biosciences) and FlowJo. Lymphocytes were identified based on their scatter patterns, and CD3+, CD8−, and CD4+ cells were considered to be CD4 T cells, while CD3+, CD8+, and CD4− cells were considered to be CD8 T cells. The CD4+ and CD8+ T cells were subsequently gated for cells positive for the respective cytokines.

### Statistical analysis

All of the presented data are expressed as the means ± standard deviation. Student’s *t*-test was used to compare the variance using Microsoft Excel, and the differences were considered significant at a probability values less than 0.05.

## Supplementary information

Table S1, Figure S1-S3

## Data Availability

All data generated or analysed during this study are included in this published article (and its Supplementary Information Files).
