# Supplementary material for: Potential of recombinant Mycobacterium paragordonae expressing HIV-1 Gag as a prime vaccine for HIV-1 infection
Source: Sci Rep. 2019 Oct 29;9:15515. doi: 10.1038/s41598-019-51875-6 (PMC6820866; doi:10.1038/s41598-019-51875-6)
Supplement: Supplementary file 1 — Table S1, Figure S1-S3 [file 41598_2019_51875_MOESM1_ESM.doc]

Supplementary Information

**Potential of recombinant *Mycobacterium paragordonae* expressing HIV-1 Gag as a prime vaccine for HIV-1 infection**

Byoung-Jun Kim, Bo-Ram Kim. Yoon-Hoh Kook and Bum-Joon Kim*

Department of Microbiology and Immunology, Biomedical Sciences, Liver Research Institute and Cancer Research Institute, College of Medicine, Seoul National University, Seoul, Korea

*Author for correspondence: Bum-Joon Kim, phD,

E-mail: [kbumjoon@snu.ac.kr](mailto:kbumjoon@snu.ac.kr)

**Supplementary Table S1.** Cytokine levels, as determined by ELISA, in vitro stimulated splenocytes with p24 from each immunized group of mice (five mice/group). Data are representative of two independent experiments. Means ± SD are shown.

| Groups | TNF-α (pg/ml) | | IFN-γ (pg/ml) | | IL-2 (pg/ml) | | IL-10 (pg/ml) | | IL-12 (pg/ml) | |
| --- | --- | --- | --- | --- | --- | --- | --- | --- | --- | --- |
|  | Day 1 | Day 3 | Day 1 | Day 3 | Day 1 | Day 3 | Day 1 | Day 3 | Day 1 | Day 3 |
| No treat | 105.88 ± 5.27 | 159.86 ± 24.44 | 665.17 ± 163.38 | 1663.71 ± 37.70 | 7.02 ± 0.21 | 38.74 ± 12.34 | 252.10 ± 31.06 | 775.58 ± 70.00 | 89.56 ± 35.96 | 127.09 ± 18.19 |
| rBCG-p24 | 507.64 ± 80.16 | 539.18 ± 60.89 | 33083.74 ± 4550.08 | 23140.62 ± 12387.19 | 19.97 ± 5.23 | 67.74 ± 10.66 | 341.18 ± 87.84 | 1304.58 ± 246.61 | 316.43 ± 73.23 | 415.07 ± 78.71 |
| rMpg-p24 | 567.53 ± 32.71 | 581.35 ± 14.71 | 47873.39 ± 6470.82 | 90989.02 ± 18664.35 | 23.64 ± 5.28 | 130.09 ± 5.48 | 269.26 ± 23.86 | 823.50 ± 129.85 | 470.88 ± 68.05 | 560.83 ± 41.41 |
| rBCG-p24 + DNA | 509.06 ± 23.78 | 548.22 ± 30.81 | 44937.14 ± 9745.28 | 47836.96 ± 4517.50 | 27.04 ± 16.85 | 85.21 ± 24.46 | 381.24 ± 106.04 | 2087.05 ± 285.95 | 454.63 ± 43.80 | 529.52 ± 4.36 |
| rMpg-p24 + DNA | 608.99 ± 19.43 | 652.75 ± 67.63 | 52672.41 ± 877.25 | 99481.58 ± 19073.64 | 27.63 ± 3.03 | 109.61 ± 35.35 | 296.44 ± 41.78 | 1646.43 ± 167.52 | 478.43 ± 24.68 | 608.46 ± 78.67 |

**Supplementary Figure S1.** The growth curve rMpg-p24 strain in 7H9 broth supplemented with ADC and 100 μg/ml of kanamycin. In the case of wild-type Mpg culture, kanamycin was excluded from 7H9 broth. To establish the growth curve, culture aliquots were taken at each time point and the OD_600_ was measured.


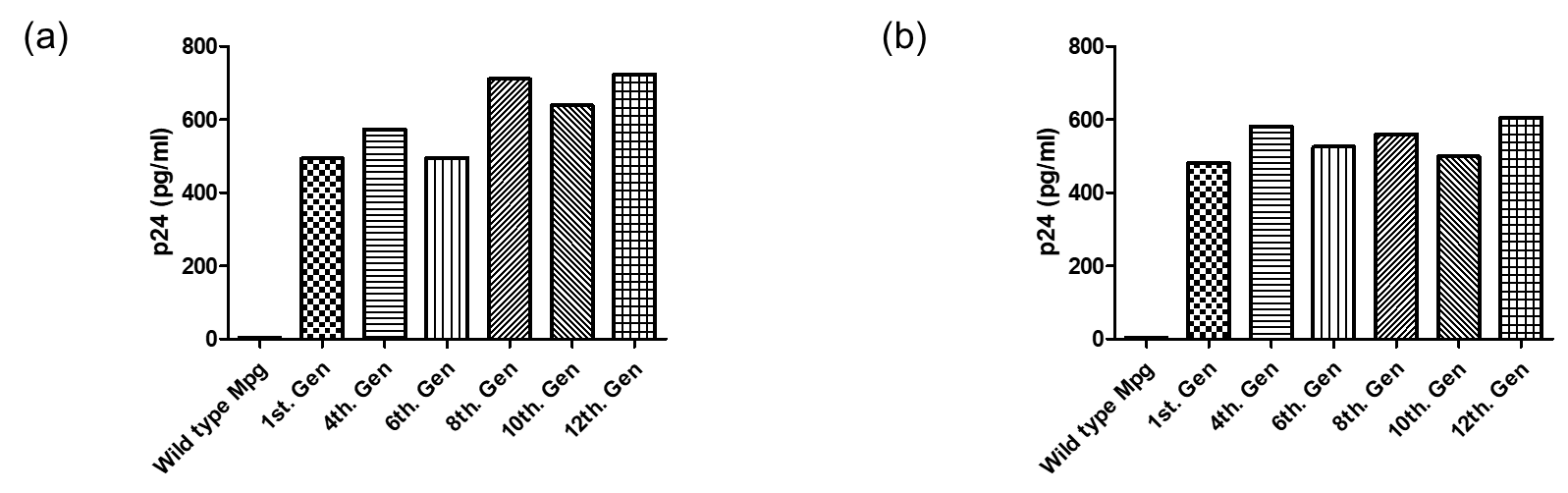


**Supplementary Figure S2.** Stability confirmation of p24 expression in rMpg-p24 strain passaged on 7H10 agar plate (a) with kanamycin or (b) without kanamycin by p24 ELISA.

1. Anti-p24


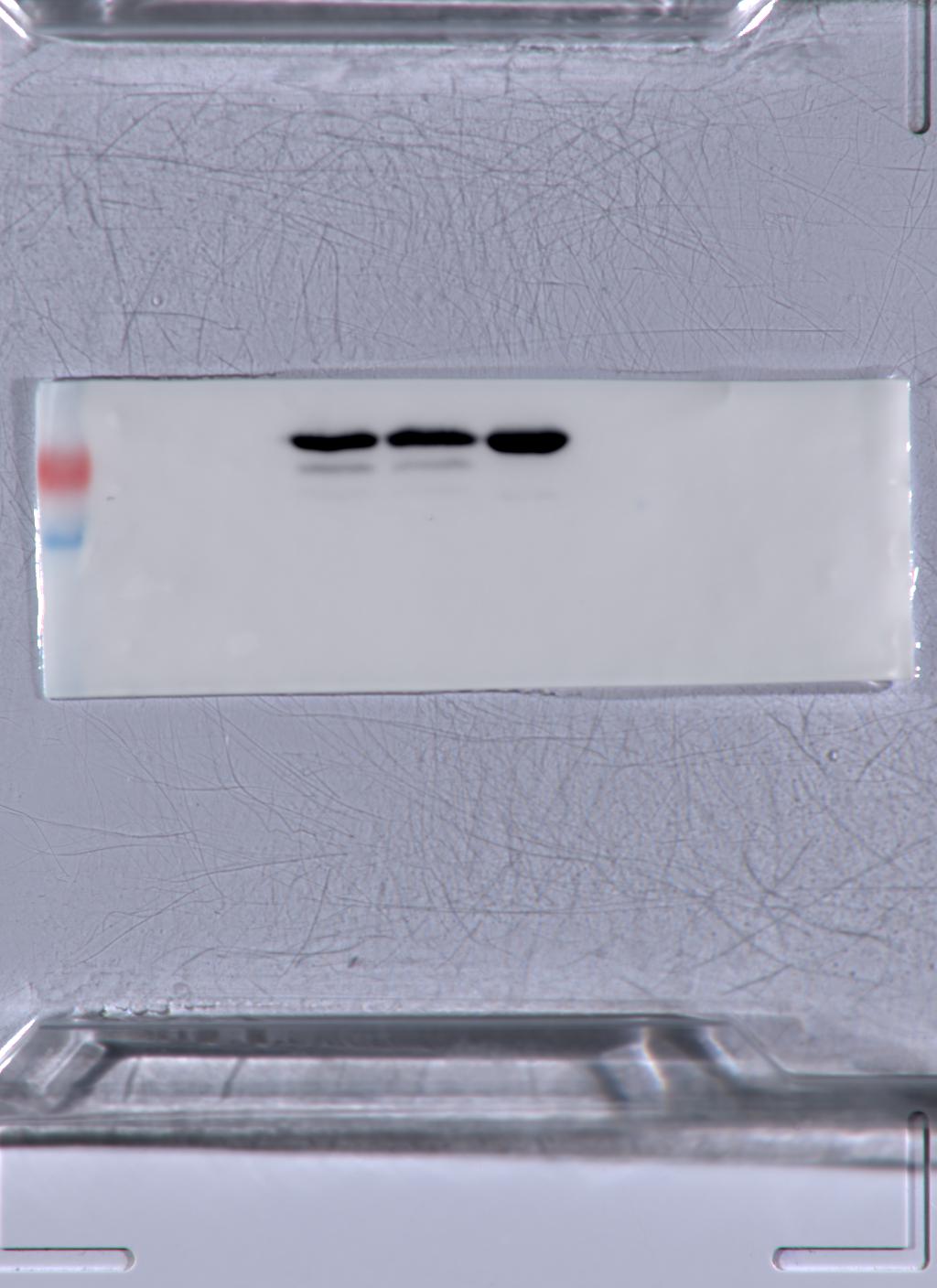


1. Anti-Hsp65


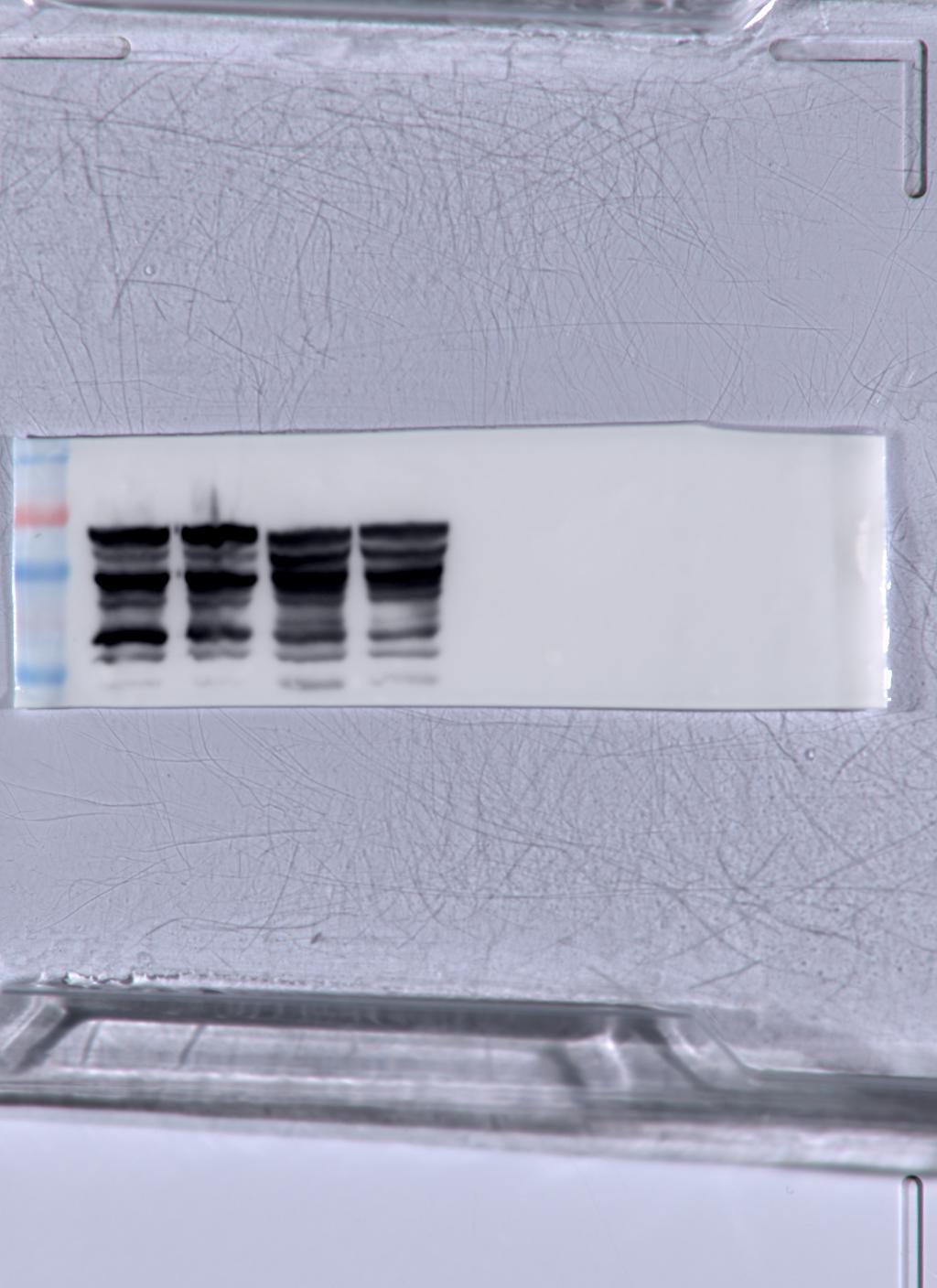


**Supplementary Figure S3.** Uncropped, full length original blots of cropped image of Fig. 2a presented in the manuscript. The blots were performed with the (a) Anti-p24 and (b) Anti-Hsp65 antibodies.
